# POSTTRAUMATIC GROWTH: REFRAMING JOB MARKET CHALLENGES AND LESSONS LEARNED

**DOI:** 10.1093/geroni/igae098.1526

**Published:** 2024-12-31

**Authors:** Sara Bybee

**Affiliations:** University of Utah College of Nursing, Salt Lake City, Utah, United States

## Abstract

In this presentation, I will use the phenomenon of posttraumatic growth—the positive psychological change that occurs as a result of the struggle with a highly stressful or traumatic event—to describe my experience searching for, applying, and obtaining a postdoctoral fellowship and later, a faculty position at an R1 institution. The five domains of posttraumatic growth (personal strength, priorities, new possibilities, relationships, and spiritual/existential change) will serve as guideposts as I discuss challenges I have faced, strategies I recommend, and lessons learned from my experiences in the job market. More specifically, I will share tips for identifying open positions, giving job talks and attending campus visits, questions that are important to ask yourself and your potential employer, and what qualities to look for when identifying possible mentors. I will also discuss professional development activities that have been pivotal in my growth, how I used what I learned from these activities to advance my career, and how I built a supportive network both within my institution, as well as across institutions. In sum, this presentation will provide attendees an opportunity to hear about applying and obtaining both postdoctoral fellowships and faculty positions, as well as both positive and negative experiences navigating the job market.

